# Comparison of Outcomes of Early and Delayed Laparoscopic Cholecystectomies in UK Clinical Practice: A Systematic Review

**DOI:** 10.7759/cureus.90940

**Published:** 2025-08-25

**Authors:** Abdaal Munir, Abdulrahman M Osman, Antonio Galluci, Mansoor Ahmed, Shakeel Ahmed, Sheila O'Neill, Conor Magee

**Affiliations:** 1 General Surgery, Wirral University Teaching Hospital NHS Foundation Trust (WUTH), Wirral, GBR; 2 Pediatric Surgery, Children's Health Ireland, Crumlin, IRL

**Keywords:** acute (mesh), cholecystectomy, cholecystitis, delayed cholecystectomy, early cholecystectomy, emergency surgery, laparoscopic (mesh), patient readmission

## Abstract

Introduction: Emergency surgical services in the UK face rising pressures, and early laparoscopic cholecystectomy (ELC) is inconsistently implemented despite National Institute for Health and Care Excellence (NICE) and the World Society of Emergency Surgery (WSES) recommendations. Limited emergency theatre capacity and scheduling bottlenecks contribute to variability. This study aimed to evaluate clinical outcomes and system impact of ELC versus delayed laparoscopic cholecystectomy (DLC) in the UK.

Methods: A systematic review of UK-based studies (2000-2025) was conducted using PubMed, Europe PubMed Central (PMC), ScienceDirect, and Cochrane Library. Eleven retrospective cohort studies were included, comprising a total of 97,574 patients who underwent laparoscopic cholecystectomy (LC). The included studies examined ELC (within 72 hours of admission) with DLC or conservative management. Outcomes assessed included conversion to open surgery, complications, length of stay (LOS), and 30-day readmissions. Cost savings were estimated using NHS reference data (£465 per inpatient bed-day).

Results: ELC was associated with reduced LOS (three to five vs. six to nine days) and lower 30-day readmission rates (1.6% vs. up to 31%). Conversion to open surgery was consistently lower in most ELC studies (0% to 5%), although one study reported rates as high as 24.2% in patients delayed beyond 72 hours of symptom onset. Across the 11 included UK-based studies (n=97,574), all patients were acute admissions deemed fit for surgery. Reporting of comorbidity, disease severity, and intraoperative difficulty measures was inconsistent: only two studies performed adjusted analyses for preoperative factors, comorbidity was reported in a minority, and no study used standardised severity grading. Complication rates were comparable or lower, with serious events such as bile duct injuries and bile leaks being rare. One large national study reported bile duct injury rates of 0.8% in the early group compared to 1.8% in delayed surgery. Some studies noted slightly higher intraoperative drain use or minor bile leaks in early cases, but without increased LOS. Estimated cost savings ranged from £1,000 to £2,000 per patient, equating to £14-28 million annually.

Conclusion: ELC is a safe, effective, and cost-saving approach for acute calculous cholecystitis (ACC) in the NHS. It reduces hospital stays and readmissions, with minimal increase in conversion risk. Broader adoption via dedicated emergency theatre slots and “hot gallbladder” lists may improve patient outcomes and ease NHS surgical pressures.

## Introduction and background

Acute calculous cholecystitis (ACC) is a common general surgical emergency, with cholecystectomy (surgical removal of the gallbladder) being the definitive treatment. In the United Kingdom (UK), gallstone disease accounts for nearly 60,000 patient admissions annually, with more than 70,000 cholecystectomies performed each year, the majority via a laparoscopic approach (laparoscopic cholecystectomy (LC)) [1,2]. Approximately 10% to 20% of patients with symptomatic gallstones will develop ACC, making it a significant contributor to the surgical workload [3].

National and international guidelines, including the National Institute for Health and Care Excellence (NICE), the World Society of Emergency Surgery (WSES), and the Tokyo Guidelines 2018 (TG18), recommend early laparoscopic cholecystectomy (ELC), ideally as soon as possible after symptom onset or within 72 hours of admission, in appropriately selected patients [4-6]. TG18 also provides standardised diagnostic criteria and a validated severity grading system, which have been widely adopted in clinical research and practice [6]. However, despite clear guidance, the quality of care in UK hospitals varies significantly.

National audits and retrospective reviews have found that a significant proportion of patients with ACC undergo delayed or elective laparoscopic cholecystectomy (DLC), often due to limited emergency theatre capacity, workforce constraints, or institutional resource prioritisation [7]. Deferring surgery can have important consequences. Patients managed conservatively (with antibiotics or supportive care rather than surgery) face risks of recurrent symptoms, unplanned readmissions, and increased operative complexity and cost at the time of interval surgery [8].

Prior systematic reviews have demonstrated the overall safety and effectiveness of ELC compared with DLC on a global scale. However, generalising these findings to the NHS is limited by differences in healthcare structure, patient flow, and perioperative management [9]. This review is the first to examine UK-based studies exclusively, drawing attention to systemic challenges within the NHS, such as limited access to emergency theatres, variation in on-call surgical rotas, and delays in referral from acute medical units, all of which affect the timing of cholecystectomy. By integrating evidence from both large national datasets and institutional observational studies, it provides context-specific insights that are directly relevant to NHS practice and can help guide clinical decision-making, inform policy planning, and improve theatre scheduling. 

In the context of this review, ELC is defined, where reported, as a cholecystectomy performed within 72 hours of hospital admission for acute cholecystitis. In contrast, DLC generally refers to surgery performed after a cooling-off period, typically ≥6 weeks following the index presentation. However, it is important to note that the exact definition of “early” varied across studies, ranging from procedures within 72 hours to up to seven to 10 days after symptom onset. Where possible, these definitions were standardised for consistency in reporting throughout this review.

## Review

Methods 

This systematic review was conducted in accordance with the Preferred Reporting Items for Systematic Reviews and Meta-Analyses (PRISMA) 2020 guidelines [10].

The aim of the review was to assess the clinical outcomes associated with ELC versus DLC in adult patients presenting with ACC in the United Kingdom. The central research question guiding this study was: In adult patients presenting with ACC in the UK, does ELC compared to DLC result in reduced postoperative complications, shorter hospital stays, and improved cost-effectiveness? To address this, the Population, Intervention, Comparison, Outcome (PICO) framework was used. The population of interest included adults aged 18 years and older with a diagnosis of ACC. ELC was defined as surgery performed within 10 days of symptom onset or within 72 hours of hospital admission. This definition aligns with practical timelines for emergency surgical care in the UK and is supported by the 2020 WSES guidelines, which recommend early intervention ideally within seven days but acknowledge that surgery within 10 days remains acceptable when delays are unavoidable [5]. In practice, most UK NHS hospitals aim to complete ELC within this window.

Some included studies reported a small number of patients who underwent preoperative endoscopic retrograde cholangiopancreatography (ERCP) or percutaneous cholecystostomy. However, these interventions were not consistently applied across all studies and were not the primary focus of this review. Where these data were clearly documented, they were included narratively to ensure transparency. Due to inconsistent reporting and lack of stratified outcomes, these subgroups were not incorporated into the pooled analysis comparing ELC and DLC.

Therefore, the review focused on surgical timing comparisons rather than guideline-based severity classification. However, many studies in this review did not consistently report the timing from symptom onset or used retrospective data, making this variable difficult to standardise. As a result, timing from hospital admission was used where symptom onset data were unavailable or unreliable, allowing for more consistent comparisons across studies. Primary outcomes assessed included postoperative complications and length of hospital stay. Secondary outcomes included readmission rates, conversion to open surgery, the need for re-intervention, and overall cost-effectiveness.

Eligibility Criteria

Eligible studies included randomised controlled trials (RCTs) and observational studies that were conducted in the UK or clearly specified a UK population. Studies were included if they involved adult patients (≥18 years) with ACC who underwent either ELC or DLC and if they reported at least one of the relevant outcomes. Only articles published in English were considered. Studies were excluded if they were case reports or case series or if they did not specify the timing of surgery or outcomes of interest.

Additional exclusions included studies focusing on acalculous cholecystitis, open cholecystectomy as the primary intervention, or cholecystectomy performed for unrelated indications such as gallbladder polyps or biliary dyskinesia. Studies involving pediatric populations or those reporting on pregnant women were excluded. Studies not specific to the UK were also excluded. Regarding the severity of cholecystitis and patient comorbidities, no restrictions were applied during study selection because these factors were not consistently reported across the included studies. Where individual studies provided data on severity grading or comorbidity indices (e.g., American Society of Anesthesiologists (ASA) physical status), these were extracted and summarised separately in the results.

To identify eligible studies, we applied filters where available within each database to limit results to English-language articles published after the year 2000. However, not all databases supported these filters. In those cases, we manually reviewed titles, abstracts, and full texts to exclude studies published before 2000 or not written in English. This manual curation ensured alignment with the predefined eligibility criteria. A comprehensive search of multiple databases was conducted, including PubMed, Medline, ScienceDirect, Cochrane Library, and Europe PubMed Central (PMC). This was completed between 25 to 27 April 2025.

The search strategy was structured around four key concepts. For ACC, keywords included “acute cholecystitis” and “cholecystitis, acute.” For laparoscopic intervention, terms such as “laparoscopic cholecystectomy” and “laparoscopy” were used. To capture timing, searches included “early,” “delayed,” “urgent,” and “immediate.” Lastly, UK-specific terms such as “United Kingdom,” “England,” “Scotland,” “Wales,” and “NHS” ensured geographical relevance. The full search strategies used across different databases are summarised in Table 1.

**Table 1:** Search strategy I/E: inclusion/exclusion; Medline: Medical Literature Analysis and Retrieval System Online; PMC: PubMed Central

Database/Register	Search strategy	Filters applied	Number of studies	Date
PubMed/MEDLINE	( "Cholecystitis, Acute"[MeSH Terms] OR "acute cholecystitis"[Title/Abstract] OR "cholecystitis"[Title/Abstract] ) AND ( "Cholecystectomy, Laparoscopic"[MeSH Terms] OR "laparoscopic cholecystectomy"[Title/Abstract] OR "laparoscopy"[Title/Abstract] ) AND ( "early"[Title/Abstract] OR "delayed"[Title/Abstract] OR "timing"[Title/Abstract] OR "urgent"[Title/Abstract] OR "immediate"[Title/Abstract] ) AND ( United Kingdom[Affiliation] OR England[Affiliation] OR Scotland[Affiliation] OR Wales[Affiliation] OR "UK"[Title/Abstract] OR "NHS"[Title/Abstract]	2000 onwards studies, English only	56	25/04/2025
Europe PMC	("acute cholecystitis") AND ("laparoscopic cholecystectomy") AND ("early" OR "delayed" OR "timing" OR "urgent" OR "immediate") AND ("UK" OR "United Kingdom" OR "England" OR "Scotland" OR "Wales" OR "NHS")	None	866	26/04/2025
ScienceDirect	"acute cholecystitis" AND "laparoscopic cholecystectomy" AND (early OR delayed OR urgent OR timing) AND ("United Kingdom" OR "UK")	None	400	27/04/2025
Cochrane Library	("acute cholecystitis")AND ("laparoscopic cholecystectomy") AND (early OR delayed OR urgent OR timing OR immediate)AND ("United Kingdom" OR UK OR NHS OR England OR Scotland OR Wales)	None	3	27/04/2025

Screening and study selection

All retrieved studies were imported into a reference management tool, Rayyan (Rayyan Systems Inc., Cambridge, MA), software for systematic screening [11]. Four reviewers were involved in the screening and study selection process. For a study to be included or excluded, a decision in favour by two assigned reviewers was required. In cases of disagreement, conflicts were resolved by discussion, and if consensus could not be reached, a senior reviewer adjudicated the final decision.

The methodological quality and risk of bias of the included studies were assessed by evaluating factors such as study design, patient selection, comparability of groups, and reporting of outcomes. Two reviewers independently assessed the studies, and any disagreements were resolved by discussion and consensus. The overall quality of evidence was summarised narratively, highlighting strengths and limitations of individual studies where relevant.

During the full-text screening phase, two independent reviewers applied a standardised checklist to evaluate key domains, including the representativeness of cohorts, comparability of groups, and adequacy of outcome reporting. This parallel process ensured methodological consistency across studies. Given that most included studies were observational cohorts, selection bias, particularly around how patients were allocated to early versus delayed surgery, was a recurrent limitation. Limited demographic data and a lack of clear matching between ELC and DLC groups were noted across several studies. This systematic review was not prospectively registered on the International Prospective Register of Systematic Reviews (PROSPERO).

Results

**Figure 1:**
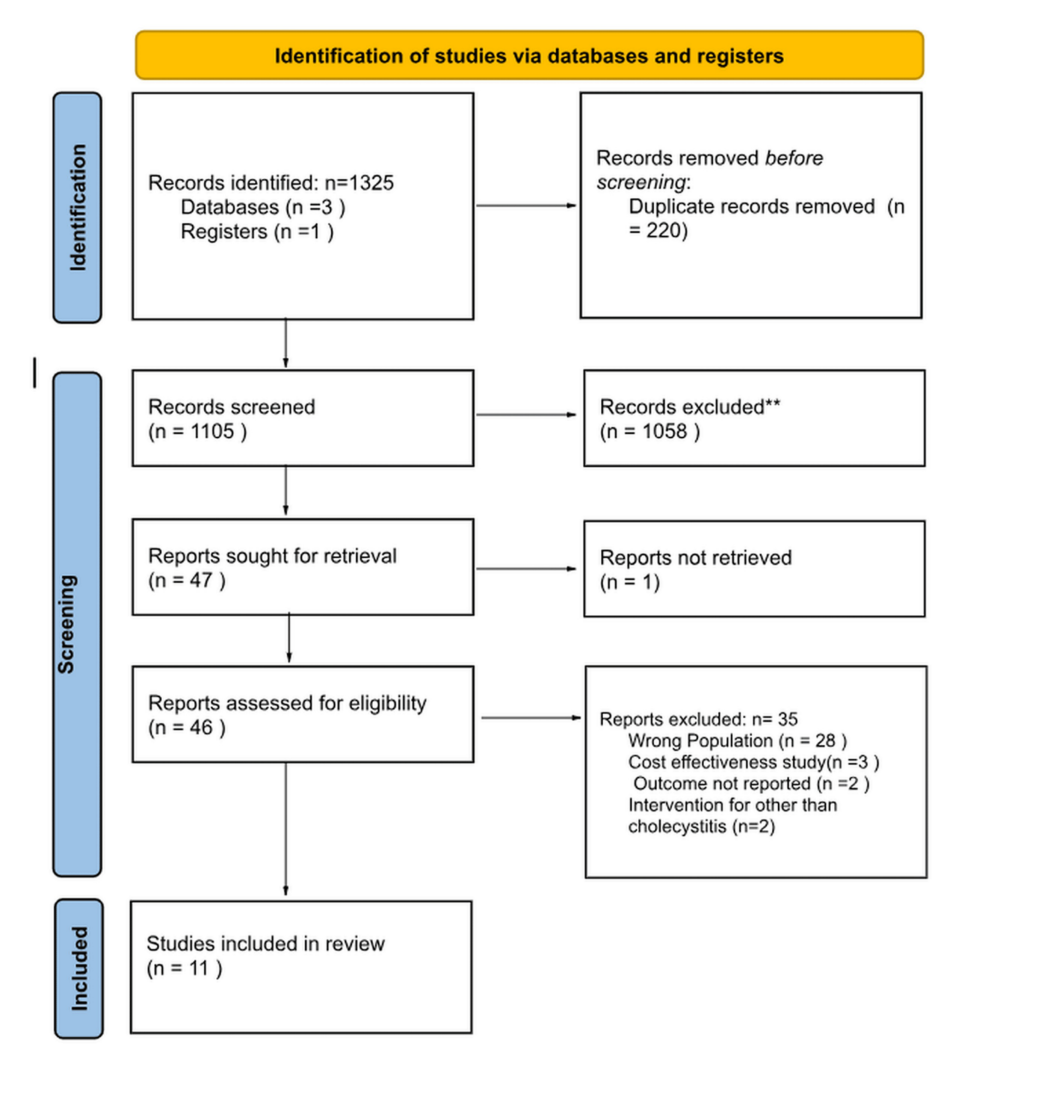
PRISMA flow diagram represents the study selection process PRISMA: Preferred Reporting Items for Systematic Review and Meta-Analysis

The review was conducted in accordance with the PRISMA guidelines [10], as illustrated in Figure 1. A total of 11 cohort studies were included in the analysis.

Data Extraction and Quality Appraisal

Two reviewers independently extracted data using a structured template and assessed study quality with the Newcastle-Ottawa Scale (NOS) [12]. A minimum score of seven was used as the threshold for inclusion. Any disagreements were resolved through consensus. This is shown in Table 2. We assessed heterogeneity based on study design, patient demographics, timing of intervention, and outcome definitions. 

**Table 2:** Quality appraisal of the included cohort studies Note: NOS permits four stars (*) for selection, two for comparability, and three for outcome. Total scores range between 0 and nine. NOS: Newcastle Ottawa Scale

Study	Selection	Comparison	Outcome	Overall
Saeb-Parsy et al., 2006. [13]	⭐⭐⭐⭐	⭐⭐	⭐⭐⭐	9 out of 9
Young et al., 2010 [14]	⭐⭐⭐⭐	⭐⭐	⭐⭐⭐	9 out of 9
Lucocq et al., 2022 [15]	⭐⭐⭐⭐	⭐⭐	⭐⭐⭐	9 out of 9
Bekheit et al., 2023 [16]	⭐⭐⭐⭐	⭐⭐	⭐⭐⭐	9 out of 9
Wiggins et al., 2019 [17]	⭐⭐⭐⭐	⭐⭐	⭐⭐⭐	9 out of 9
Hadad et al., 2007 [18]	⭐⭐⭐	⭐⭐	⭐⭐⭐	8 out of 9
Singhal et al., 2006 [19]	⭐⭐⭐⭐	⭐	⭐⭐⭐	8 out of 9
Down et al., 2010 [20]	⭐⭐⭐⭐	⭐	⭐⭐⭐	8 out of 9
Farooq et al., 2009 [21]	⭐⭐⭐⭐	⭐⭐	⭐⭐⭐	9 out of 9
Vithayathil et al., 2025 [22]	⭐⭐⭐⭐		⭐⭐⭐	7 out of 9
Stephens et al., 2010 [23]	⭐⭐⭐⭐		⭐⭐⭐	7 out of 9

**Figure 2:**
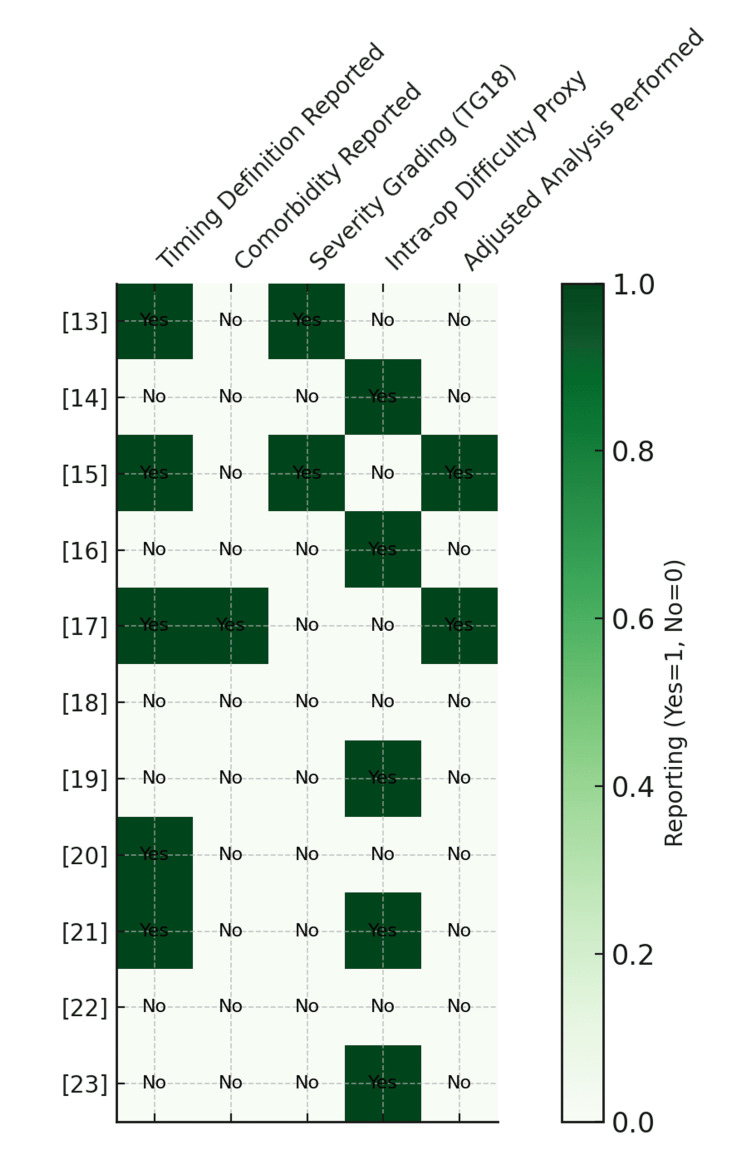
Matrix of reported comorbidity, severity, intra-operative difficulty, and analytical adjustments in included UK studies Timing definition: a clear operational definition of “early” versus “delayed” laparoscopic cholecystectomy; Comorbidity measures: American Society of Anaesthesiologists (ASA) classification, Charlson Comorbidity Index (CCI); Standardised severity grading: Tokyo Guidelines (TG18) severity classification; Intra-operative difficulty proxy: conversion to open surgery, subtotal cholecystectomy, intra-operative drain use; Adjusted analysis performed: statistical adjustment for preoperative factors such as age, comorbidity, or laboratory findings. This figure has been created by the authors.

Data were extracted on study characteristics, sample size, setting, timing of surgery, and reported outcomes, including length of stay (LOS), readmissions, and complication rates. We recorded definitions of key outcomes, including complication severity and timing of ‘early’ surgery, as reported by each study. Variations in these definitions were addressed narratively in the synthesis, given the lack of standardisation across studies. For each study, we extracted any reported measures of comorbidity (e.g., ASA, Charlson Comorbidity Index), disease severity (e.g., Tokyo Guidelines), and intraoperative difficulty (e.g., conversion to open, drain use, subtotal cholecystectomy). We also recorded whether analyses were adjusted for preoperative factors. These variables were summarised in a reporting matrix to assess between-group comparability (Figure 2).

Heterogeneity among the included studies was evident and primarily stemmed from several methodological and clinical differences. These included variability in the definitions and timing thresholds for ELC versus DLC (e.g., <72 hours, <5 days, <10 days), differences in patient selection criteria, and the format of outcome reporting (e.g., means vs. medians, unreported or incomplete statistics). Additionally, some studies included patients who had undergone adjunct interventions such as preoperative ERCP or percutaneous cholecystostomy, while others excluded them.

Population Characteristics and Group Comparability

All included studies involved patients admitted acutely with a diagnosis of ACC who were considered fit for surgery by the treating surgical team. The decision to proceed with ELC or to delay the procedure (DLC) was made on the basis of clinical judgement and the availability of theatre time, rather than through random allocation.

The extent to which baseline risk factors were reported varied widely across the studies. Only two studies, Wiggins et al. [17] and Lucocq et al. [15], adjusted analyses for preoperative characteristics such as patient age and the presence of co-existing medical conditions. Measures of comorbidity, for example, the ASA physical status classification system and the Charlson Comorbidity Index, were documented in only a small proportion of the included cohorts. None of the studies applied a standardised disease severity grading system such as the Tokyo Guidelines. Indicators of intra-operative difficulty, including conversion to open surgery, the need for intra-operative drains, or the use of subtotal cholecystectomy, were also reported inconsistently.

A visual summary of reporting across these domains is presented in Figure 2. This matrix highlights that, while ELC generally showed favourable outcomes, between-group comparability is limited by incomplete severity/comorbidity reporting, particularly in unadjusted single-centre studies. These differences may partially explain outcome variation between studies.

Baseline Characteristics

Across the included UK studies, baseline demographic and clinical characteristics were variably reported. The median age of patients ranged from 50 to 58 years among studies reporting this data, with a slight female predominance across most cohorts (59% to 65%). ASA physical status was reported in only three studies. Saeb-Parsy et al. [13] found no significant difference in ASA grades between early and delayed groups, while Down et al. explicitly excluded ASA grade IV-V patients from emergency LC, thereby selecting lower-risk patients for early surgery (Table 3). The Wiggins et al. [17] national dataset was adjusted for comorbidity using the Charlson Comorbidity Index instead of ASA scores, as ASA data were unavailable in the Hospital Episode Statistics (HES) database. None of the included studies reported outcomes stratified by ASA grade. Reporting of other baseline parameters, such as BMI, gallbladder wall thickness, or inflammatory markers, was inconsistent and often incomplete, limiting cross-study comparability.

**Table 3:** Baseline characteristics of the included studies ASA: American Society of Anesthesiologists

Study	Sample size	Median age (years)	Female (%)	Male (%)	ASA grade reported
Saeb-Parsy et al., 2010 [13]	423	54	63	37	Yes
Young et al., 2010 [14]	1710	52	61	39	NR
Lucocq et al., 2022 [15]	811	55	64	36	Yes
Bekheit et al., 2023 [16]	47558	57	62	38	NR
Wiggins et al., 2019 [17]	43870	56	60	40	NR
Hadad et al., 2007 [18]	1385	53	59	41	NR
Singhal et al., 2006 [19]	119	50	65	35	NR
Down et al., 2010 [20]	326	58	62	38	Yes
Farooq et al., 2009 [21]	239	51	61	39	NR
Vithayathil et al., 2023 [22]	105	54	64	36	NR
Stephens et al., 2010 [23]	627	56	63	37	NR

Summary of Findings

This systematic review synthesises data from 11 UK-based studies comparing ELC and DLC in the management of ACC. The included studies range from single-centre observational cohorts to national database analyses, with sample sizes spanning from 105 to over 43,000 patients. The outcomes across the studies are highlighted in Table 4.

**Table 4:** Summary of the included studies LOS: length of stay; ELC: early laparoscopic cholecystectomy; DLC: delayed laparoscopic cholecystectomy; AC: acute cholecystectomy; † Median LOS of 0 likely reflects administrative discharge coding, not actual same-day discharge; ‡ Timing definitions not reported in the original study; § Conversion rates not reported.

Study	Place of study	Design	Sample size	Comparison	Timing definition (ELC vs. DLC)	Conversion rate ELC % (n=)	Conversion rate DLC % (n=)	Complication rate % (n)	Length of stay median (days)	Readmission rate % (n=)	Intervention details	Key findings
Saeb-Parsy et al., 2010 [13]	Hinchingbrooke Hospital	Observational	423	ELC vs. DLC	ELC: <5 days; DLC: NR	0	0.3	ELC:13.1; DLC:7.3	ELC median 4 (2,6) DLC median 0 (0,2)†	ELC: 1.6 (2) DLC: 3.3 (10)	Standard ELC/DLC	ELC is safe, has low conversion and complications
Young et al., 2010 [14]	St.James University Hospital, Leeds	Retrospective	1,710 (AC elective: 161; Acute: 125)	Acute vs. elective cholecystectomy for biliary symptoms	NR‡	10 (42)	3 (42)	ALC:4 (19), Elective: 3 (41)	ALC median: 6 (1-34), Elective: 2(0-34)	NR	Retrospective comparison	Favourable for Acute LC, Age over 65 years, Male gender, cholecystitis, and thicker gallbladder wall, p = 0.001, associated with higher conversion rates
Lucocq et al., 2022 [15]	Ninewells Hospital, Dundee	Retrospective	811	ELC vs. DLC	NR‡	1.3 (3)	2.7 (15)	ELC: 4.4 (10) DLC: 3.1(17)	ELC: 5; DLC: 7	ELC: 13.2 (30); DLC: 8.5 (47)	Population analysis	ELC had higher perioperative morbidity but lower readmissions. Higher median operative time for ELC when compared to DLC
Bekheit et al., 2023 [16]	Scottish National Health Service	Retrospective	47,558	ELC vs. conservative treatment at index presentation	NR‡	NR§	NR§	NR	ELC: 4 Conservative: 3	NR	Multicentre analysis	ELC reduced LOS and mortality by 1.6% compared to conservative 2.4 %
Wiggins et al., 2019 [17]	England (National)	Population database	43,870	ELC 0–3 vs. 4–7 vs ≥8 days	ELC: <3 days; DLC: ≥6 weeks	0-3 days; 2.8 vs. 4-7 days; 4.0 vs. >8 days; 4.7	NR§	0-3 days; 1.1 vs. 4-7 days; 1.5 vs. >8 days; 1.9	0-3 days: 3 vs.4-7 days: 3 vs. >8 days; 4	NR	National audit	Earlier surgery had fewer complications, reduced bile duct injury rates in the 0-3 days group, 0.8 when compared to the more than 8 days group, 1.8, p <0.001
Hadad et al., 2007 [18]	Nine Wells Hospital, Dundee, UK	Observational	1,385	ELC with outcomes analysis on delay from symptom onset	NR‡	24.2	NR§	NR	NR	NR	Consultant-led index surgery	ELC is feasible and safe, but the delay from symptoms to procedure increases the conversion to open rates
Singhal et al., 2006 [19]	The Princess Royal Hospital, Kent, UK	Prospective	119	ELC for gallstone-related disease	NR‡	5.04	NR§	0.06	3.1	0.8	Index admission LC	ELC had fewer complications, shorter LOS; all patients with AC were operated on within 96 hours
Down et al., 2010 [20]	Cambridge University Hospitals, NHS	Retrospective	326	Emergency vs. Elective	ELC: <72 hrs; DLC: ≥6 weeks	0	0	7.5	ELC 1; DLC 0.3	8.8	Comparison elective/emergency LC	No significant difference in complications
Farooq et al. 2009. [21]	Yeovil District Hospital NHS Foundation Trust, Yeovil	Retrospective	239	Group I: ELC within 3 days vs. Group II: ELC after 3 days	NR‡	Group I: 7, Group II: 10	NR§	NR	Group I: 2 vs. Group II: 3	6 in both groups	Hospital LOS audit	ELC within 3 days has shorter LOS, Median Operative time is similar in both groups, Lower conversion rates in Group I as compared to Group II
Vithayathil et al., 2023 [22]	University College London Hospitals, NHS Trust	Retrospective	105	ELC (7) vs. DLC (98)	NR‡	NR§	NR§	ELC: 0; DLC: 8.2	ELC: 6; DLC: 6	DLC: 25.5	Biweekly review of DLC cases	High readmission in the DLC group, with similar postoperative LOS of 2 days in both groups
Stephens et al., 2010 [23]	Nevill Hall Hospital, Abergavenny (NHH) and Royal Gwent Hospital, Newport (RGH), UK	Cohort analysis	627	ELC vs. Elective	NR‡	17.4	8.2 in elective	NR	NR	ELC: 4; DLC: 31	National patient record analysis	ELC had higher conversion rates in acute cholecystitis. Elective cholecystectomy had a high readmission rate

Key Clinical Outcomes

Outcomes were variably reported across the included studies, with only four studies providing sufficient data to calculate or extract 95% confidence intervals (CIs). As summarised in Table 4, Wiggins et al. [17] demonstrated that ELC was associated with significantly lower complication rates and reduced conversion to open surgery compared with DLC. Similarly, Saeb-Parsy et al. [13] and Lucocq et al. [15] reported higher complication and readmission rates in delayed cohorts, although findings from Down et al. [20] differed, showing higher readmissions among ELC patients, likely reflecting case-mix differences (Table 5).

**Table 5:** Key outcomes ELC: early laparoscopic cholecystectomy; DLC: delayed laparoscopic cholecystectomy; LOS: length of stay; NR: not reported

Study	Comparison	Timing definition (ELC vs. DLC)	Conversion rate ELC% (95% CI)	Conversion rate DLC% (95% CI)	Complication rate ELC% (95% CI)	Complication rate DLC% (95% CI)	Median LOS (days)	Readmission rate ELC% (95% CI)	Readmission rate DLC% (95% CI)
Saeb-Parsy et al., [13]	ELC vs. DLC	<5 days; DLC NR	0% (NR)	0.3% (0.1–1.2)	13.1% (9.7–17.5)	7.3% (5.1–10.5)	4 (ELC) vs. 0 (DLC)	1.6% (0.4–5.7)	3.3% (1.7–6.3)
Lucocq et al., [15]	ELC vs. DLC	NR	1.3% (0.4–3.7)	2.7% (1.5–4.8)	4.4% (2.4–7.8)	3.1% (1.9–5.1)	5 (ELC) vs. 7 (DLC)	13.2% (9.4–18.3)	8.5% (6.3–11.3)
Wiggins et al., [17]	ELC 0–3 vs. 4–7 vs ≥8 days	<3 days; ≥6 weeks	2.8% (2.5–3.2)	4.7% (4.1–5.3)	1.1% (0.9–1.3)	1.9% (1.6–2.3)	3 (ELC) vs. 4 (DLC)	NR	NR
Down et al., [20]	Emergency vs. Elective	<72 hrs; ≥6 weeks	0% (NR)	0% (NR)	7.5% (4.4–12.5)	NR	1 (ELC) vs. 0.3 (DLC)	8.8% (5.6–13.5)	2.8% (1.4–5.6)

Overall, these data indicate that ELC is generally associated with shorter hospital stays, lower conversion rates, and comparable or reduced complication rates. However, inconsistent reporting of statistical measures, including CIs, limited the ability to perform pooled analyses, necessitating a descriptive synthesis.

Complication Rates

**Figure 3:**
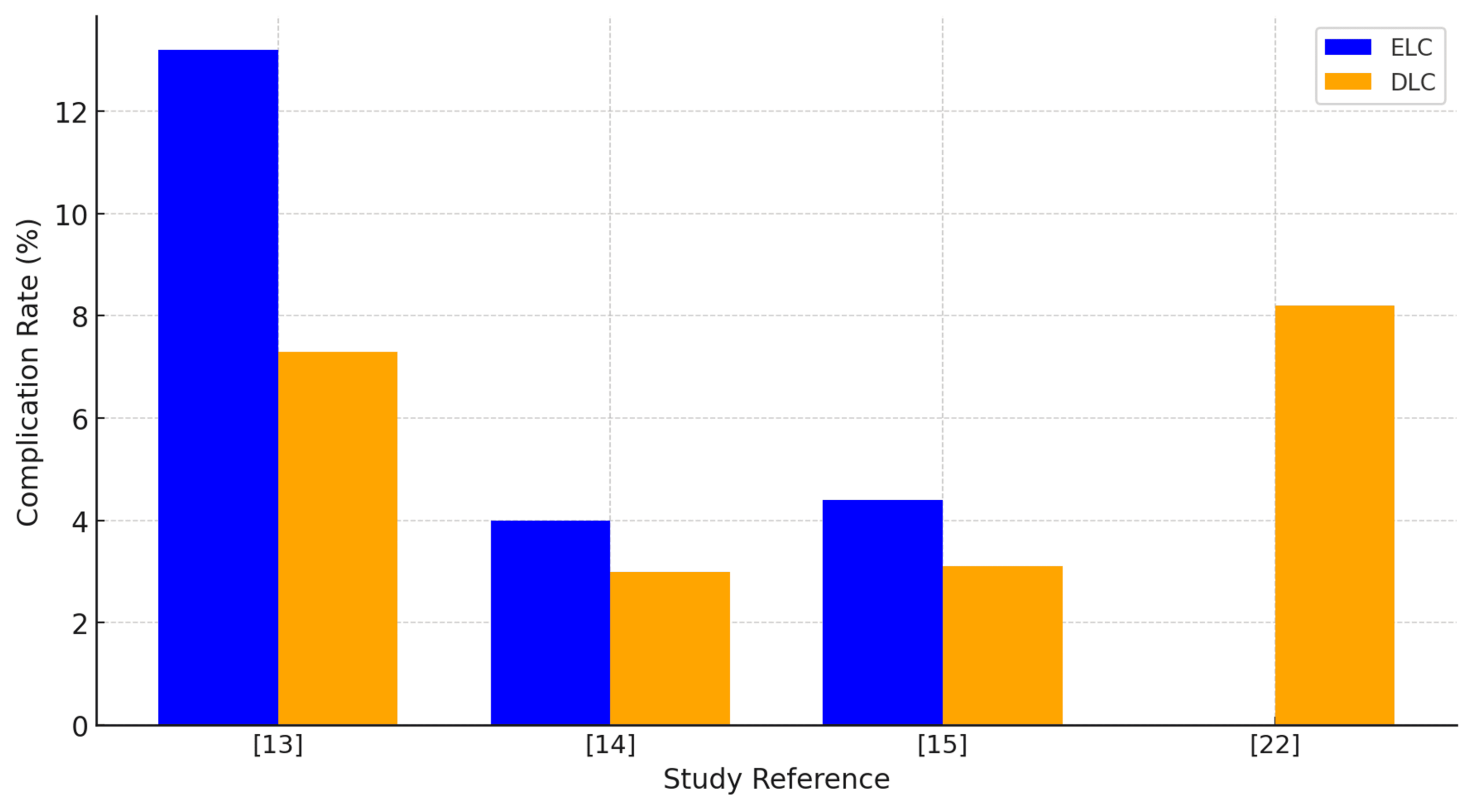
Complication rates across studies ELC: early laparoscopic cholecystectomy; DLC: delayed laparoscopic cholecystectomy This figure has been created by the authors.

Complication rates in ELC versus DLC across 11 UK studies were noted. Although data were not uniformly reported in all studies, ELC generally demonstrated equal or slightly higher complication rates in some reports, often related to the acuity of presentation, while DLC showed variability likely linked to interval complications or selection bias. National-level data from Wiggins et al. [17] indicated lower complications with early surgery. This trend is visually summarised in Figure 3, which compares complication rates across included studies.

Readmission Rates and Cost-Effectiveness

**Figure 4:**
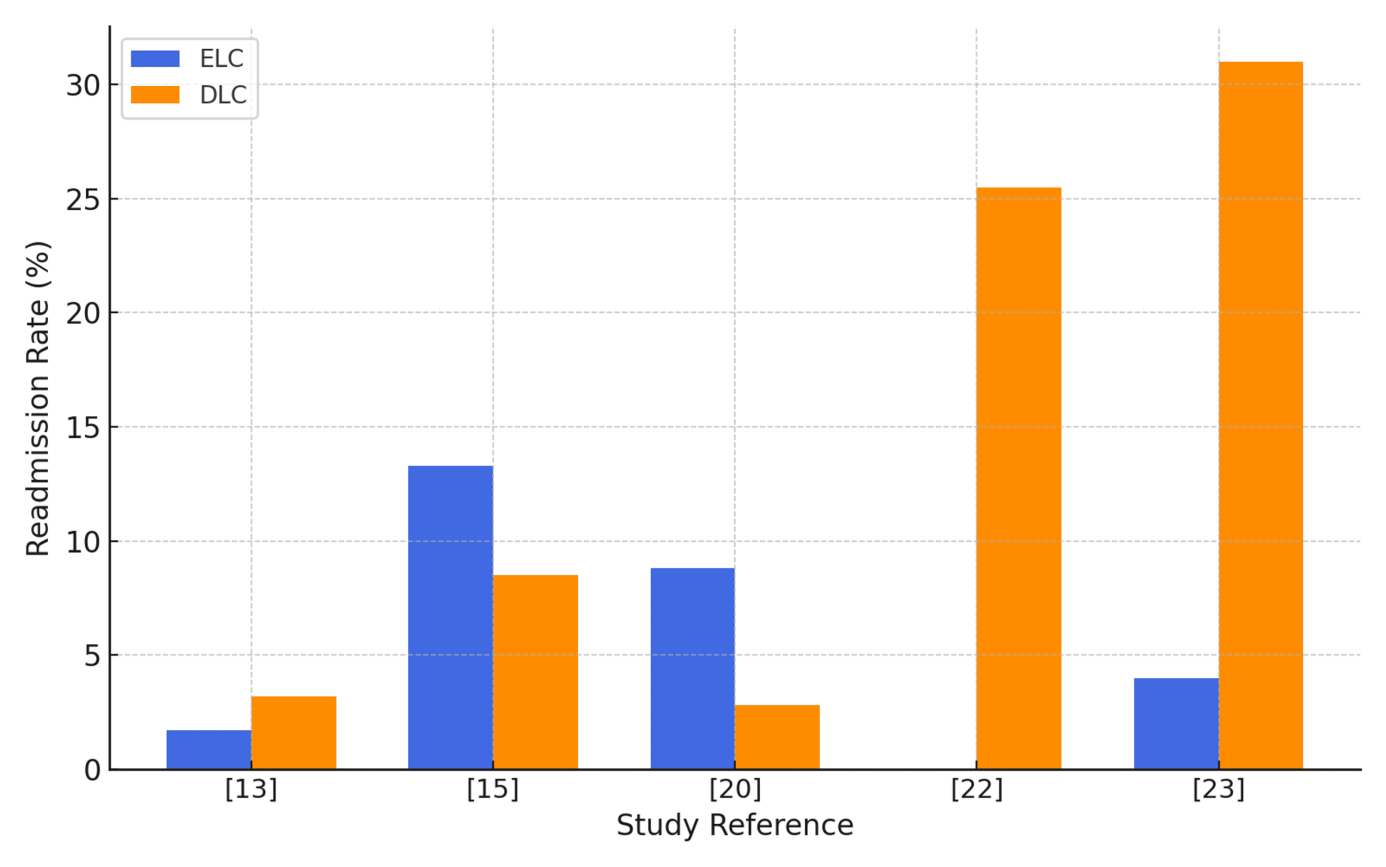
A comparison of readmission rates in ELC and DLC ELC: early laparoscopic cholecystectomy; DLC: delayed laparoscopic cholecystectomy This figure has been created by the authors.

Readmission rates were generally lower for ELC. Saeb-Parsy et al. [13] found fewer readmissions after early surgery. In contrast, Down et al. [20] reported a higher readmission rate in the early group (8.8%) compared with the delayed group (2.8%). This difference may reflect differences in patient selection, as the early cohort in that study included a higher proportion of urgent or complex cases. Figure 4 presents the readmission rates associated with ELC and DLC as reported in the included studies. DLC showed significantly higher readmission rates, with some studies reporting figures exceeding 25% to 30%, highlighting the potential burden of conservative or delayed surgical management. 

While only a minority of the included studies directly evaluated cost outcomes, the overall findings indicate that ELC is likely to reduce healthcare expenditure within the NHS. Studies such as Wiggins et al. [17] and Saeb-Parsy et al. [13] reported that performing cholecystectomy during the index admission leads to shorter total hospital stays and fewer readmissions, both of which are key drivers of reduced cost.

Conversion Rates

**Figure 5:**
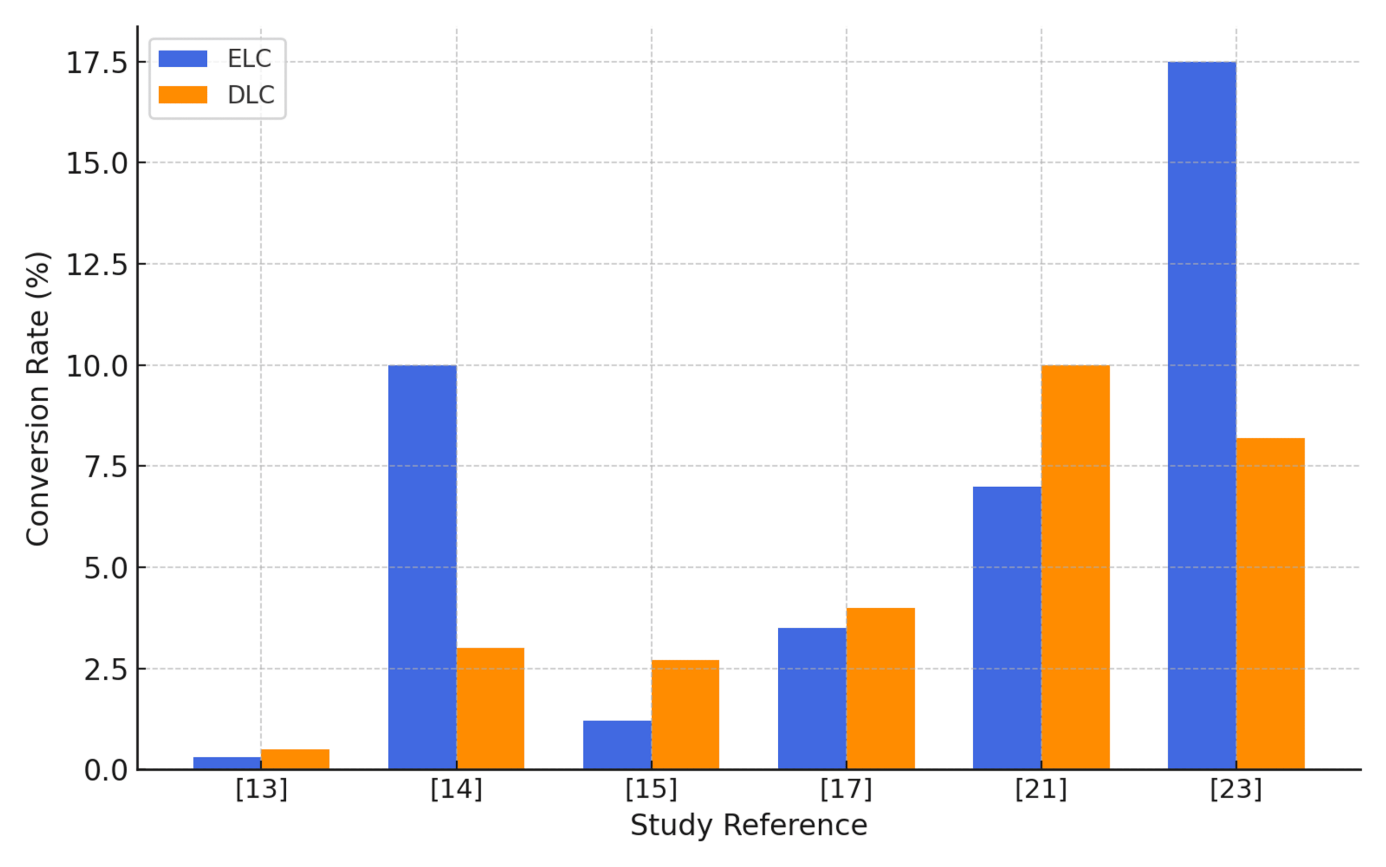
Conversion rates to open surgery ELC: early laparoscopic cholecystectomy; DLC: delayed laparoscopic cholecystectomy This figure has been created by the authors.

Conversion rates differed across studies. Two studies [14, 23] reported higher rates in early surgery groups, potentially reflecting the inclusion of more severe cases or patients with higher comorbidity. In contrast, most studies [13, 15, 17, 21] observed lower conversion rates with early intervention. Saeb-Parsy et al. [13] and Lucocq et al. [15] reported rates of 0% and 1.3% in ELC groups versus 0.3% and 2.7% in DLC groups, respectively. Wiggins et al. [17] found the lowest rate (2.8%) when surgery occurred within three days, rising to 4.7% after Day 8. Variability in patient selection, severity of cholecystitis, comorbidity profiles, and timing thresholds likely contribute to these differences (Figure 5).

Discussion

Summary of Key Findings

This systematic review of UK-based studies comparing ELC with DLC or conservative management highlights consistent advantages of early intervention. Across multiple centres and large datasets, ELC is associated with shorter hospital stays, comparable or lower complication rates, and a reduced need for conversion to open surgery. While some variation exists in patient demographics and definitions of “early”, the overall trend supports ELC as a safe and effective strategy in the UK context.

Clinical Outcomes: Complications 

While complication rates varied between studies, there was no consistent evidence that early surgery was associated with greater morbidity. Saeb-Parsy et al. [13] reported a higher overall complication rate in the early group (13.1%) compared with the delayed group (7.3%), though most were minor events; serious complications such as bile leak and the need for re-intervention were rare, occurring in 0.8% and 0% of cases, respectively. Lucocq et al. [15] also found a slightly higher perioperative morbidity with early surgery (4.4% versus 3.1%), which may reflect the selection of patients with more acute symptoms. In contrast, Wiggins et al. [17], using national audit data, demonstrated lower complication rates with earlier surgery, 1.1% in the 0- to three-day group compared with 1.9% when surgery was delayed beyond eight days. Wiggins et al. [17] also found that bile duct injury rates were lower with early surgery (0.8%) than with delayed surgery (1.8%). Saeb-Parsy et al. [13] reported similar findings for bile leak, with a rate of 0.8% in the early group and no cases requiring re-intervention. Lucocq et al. [15] noted that drains were used more often in early cases, possibly as a precaution when operating in inflamed tissue, but this did not prolong hospital stay.

Conversion Rates

The overall evidence does not support a generalised increase in conversion rates with early surgery. Only two studies [13, 22] showed higher rates in early cohorts, both of which included a greater proportion of patients with severe inflammation or higher operative difficulty. Most studies [13,15,17,21] demonstrated lower rates when cholecystectomy was performed early, consistent with the principle that operating before dense adhesions develop can reduce technical difficulty. However, the absence of standardised severity grading and inconsistent reporting of comorbidities limit our ability to determine whether differences are attributable to timing alone.

Hospital Stay, Readmissions, and Variability in Outcome Reporting

Across UK studies, LOS was consistently shorter for patients who underwent ELC. Bekheit et al. [16] reported a median LOS of four days in the ELC group compared with three days in patients managed conservatively, although the latter had higher long-term mortality. Wiggins et al. [17] found that patients operated on during the index admission had a median LOS of three days versus four days for those managed with interval surgery, the difference largely reflecting the additional hospital visit required for delayed cholecystectomy. Similarly, Saeb-Parsy et al. [13] showed that combined LOS (index admission plus any readmission) was shorter among patients who had ELC compared with those discharged and later readmitted for DLC. Collectively, these findings highlight that avoiding undue delay reduces overall hospital stay and optimises resource use.

Readmission rates showed greater variation. Several studies, including Saeb-Parsy et al. [13], Wiggins et al. [17], and Vithayathil et al. [22], reported fewer readmissions following ELC compared with DLC. In the series by Saeb-Parsy et al. [13], the difference was modest (1.6% versus 3.3%), whereas Wiggins et al. [17] demonstrated that the lowest readmission rates occurred when surgery was performed within the first three days of admission. Vithayathil et al. [22] reported the most marked difference, with no readmissions in the early cohort compared with over one-quarter of patients in the delayed group. By contrast, Lucocq et al. [15] and Down et al. [20] observed higher readmission rates among patients undergoing ELC (13.2% vs. 8.5% and 8.8% vs. 2.8%, respectively). In both cases, the ELC groups included a larger proportion of urgent or clinically complex presentations, whereas the DLC groups more often comprised lower-risk patients selected for elective surgery after their acute episode had resolved. These case-mix differences likely explain the apparent advantage of delay reported in those studies.

Interpretation of these outcomes is further complicated by variability in study methodology. The definition of “early” surgery differed widely, ranging from procedures performed within 72 hours of admission to as late as 7-10 days. Wiggins et al. [17], who defined early as <3 days, found the most favourable outcomes, while studies using broader windows often reported higher morbidity. Similarly, the definition and reporting of complications were inconsistent: some authors included only major adverse events such as bile duct injury or reoperation, whereas others reported minor complications, including wound infections or urinary tract infections. This heterogeneity complicates direct comparisons and may mask true differences between ELC and DLC.

Taken together, the weight of evidence suggests that ELC shortens overall LOS and, in most studies, reduces biliary-related readmissions. However, heterogeneity in the timing of “early” surgery and inconsistent outcome definitions limit the ability to draw uniform conclusions. Standardising reporting criteria in future studies would improve comparability and strengthen the evidence base. In this review, these methodological differences precluded a reliable meta-analysis; instead, a descriptive synthesis was undertaken to more accurately reflect the diversity of UK surgical practice.

Timing and Surgical Safety

Surgical timing is crucial to the outcome. Early cholecystectomy is most commonly defined as an operation within 72 hours of acute admission, though some define 'early' as seven to 10 days from the onset of symptoms. Wiggins et al.’s national audit showed an unequivocal tendency: the earlier the operation, the lower the complication rate and the number of conversions to open surgery [17]. This is in accordance with international recommendations (i.e., NICE and WSES) for a cholecystectomy within a week of the diagnosis of acute cholecystitis. Crucially, the historic fear of performing surgery during inflammation is being ever more seriously questioned. Only a minority of studies included operative time data, which limits the interpretation of intraoperative complexity. Lucocq et al. [15] reported longer median operative times in the ELC group, possibly reflecting more technically challenging dissection in inflamed tissues. However, Farooq et al. [21] found no significant difference in operative duration between early and delayed groups, and Young et al. [14] suggested that male gender, older age, and gallbladder wall thickness were predictive of increased difficulty, though without reporting duration. The accumulated evidence suggests that early surgery is safe and may even be beneficial in the modern era, where both laparoscopy and perioperative care are available with high standards for selected patients. That is, there may be no benefit to waiting and potentially worsening the situation with a subsequent gallstone attack or further complications while awaiting the surgery.

Strengths and Limitations

One of the main strengths of this review is the restriction to UK-based data, making the results highly relevant to practice within the NHS system. Both single-centre studies and large national registries were included, supporting generalisability. However, there are notable limitations.

A key limitation is the inconsistent reporting of critical variables across the included studies. Definitions of early and delayed cholecystectomy varied significantly, ranging from <72 hours to <10 days for early intervention, and were not consistently reported in all studies. Discrepancies in outcome reporting formats, such as means versus medians, absence of confidence intervals, and use of administrative coding (e.g., a median LOS of 0 days), complicate direct comparisons. The widespread use of “Not Reported” (NR) values, especially in large cohort studies, further limits data completeness. While explanatory footnotes were added to Table 3 to clarify these inconsistencies, the interpretability of pooled findings remains constrained. There was also inconsistent reporting of adjunct procedures such as preoperative ERCP or cholecystostomy. While some studies mentioned these interventions, they were not applied universally or reported in a stratified manner, limiting their analytical value. Including these cases without clear outcome separation would risk skewing comparisons between ELC and DLC; therefore, where reported, they were discussed narratively but excluded from aggregate analysis.

Although the TG18 provide a standardised severity grading system for acute cholecystitis, its application was limited across the studies reviewed, restricting severity-adjusted comparisons between ELC and DLC [6]. Moreover, the TG18 grading system may have limited predictive value for operative outcomes in NHS settings, where decision-making often incorporates broader variables. Another major limitation was the inconsistent reporting of key baseline parameters, including comorbidity measures, severity grading, and intraoperative difficulty indicators. Only two studies used adjusted analyses for preoperative risk factors; most were unadjusted observational comparisons. Figure 2 illustrates the heterogeneity in reporting, which limits the ability to fully account for differences between early and delayed groups and may explain some variation in outcomes.

Another important limitation is the absence of a consistent assessment for clinically relevant confounding factors such as baseline frailty, comorbidity burden, and nutritional status. Preoperative malnutrition, in particular, is an established predictor of surgical morbidity and mortality, yet none of the included studies incorporated structured nutritional assessment before cholecystectomy. Evidence from a large multicentre prospective study by Petra et al. [24] has demonstrated the diagnostic and prognostic validity of malnutrition assessment tools in surgical patients, underscoring the potential relevance of this factor when comparing outcomes between early and delayed surgery.

Finally, important unmeasured confounders, such as surgeon experience, subspeciality training, intraoperative decision-making thresholds, and institutional protocols, may also have influenced operative difficulty, complication rates, and timing of surgery. Variability in service availability across NHS trusts could similarly have impacted outcomes. Despite these caveats, these limitations do not contradict the overall direction of evidence favouring early intervention. This review was not prospectively registered on PROSPERO or any other registry. While this is a limitation for full transparency, the review adhered strictly to PRISMA 2020 guidelines. The search strategy, inclusion criteria, and data extraction process have been fully described to allow replication by other researchers.

Comparison with Previous Systematic Reviews

Previous systematic reviews, including those conducted in China and internationally, such as Gurusamy et al. (2013) [3] and Cao et al. (2015) [9], have consistently reported that patients undergoing early cholecystectomy achieve a shorter total length of hospital stay, experience fewer gallstone-related complications while awaiting surgery, and have similar or lower morbidity compared with those undergoing delayed surgery. However, these reviews combined studies from diverse health systems with varying hospital resources and surgical practices, making their findings less directly applicable to the UK context. More relevant to NHS policy and practice, our review is the first to focus exclusively on UK-based studies.

This UK-specific approach enabled us to identify systemic issues unique to the NHS, such as restricted access to emergency theatres, variation in on-call surgical rotas, and delays in referral from acute medical units, that can influence the timing of cholecystectomy. Notably, national-level analyses such as Wiggins et al. (2019) [17] and real-world observational studies like Cheruvu et al. (2015) [7] and Bekheit et al. (2023) [16] demonstrate that ELC is both feasible and beneficial within current NHS constraints. These conclusions are also supported by Palomba et al. (2021) [25], who found that incorporating both the onset of symptoms and severity grading into surgical decision-making can help optimise timing, with early intervention associated with improved outcomes. While global reviews emphasise the general advantages of early surgery, our UK-focused findings suggest that implementing early cholecystectomy in the NHS could not only improve patient care but also enhance surgical training opportunities and reduce healthcare costs. In this regard, our review addresses a critical evidence gap by providing NHS-specific data to inform evidence-based guidelines, resource allocation, and service delivery models.

Implications for UK Surgical Practice

Despite compelling evidence for early intervention, ELC is not yet universally implemented throughout the NHS. Barriers include restricted access to emergency theatres, competing with other emergency cases (urgencies), and differences among health services in workforce deployment (for example, not all hospitals offer a dedicated biliary emergency list or do not have enough staffed theatre slots for non-life-threatening emergencies). Yet a number of UK centres have shown that these difficulties may be surmounted by system redesign. For instance, Cheruvu et al. (2015) [7] and Agrawal et al. (2009) [8] demonstrated that by creating dedicated “hot gallbladder” operating lists, or systemising a “surgeon of the week” model providing dedicated attention to emergency general surgery, it was possible to significantly raise the rates of index admission cholecystectomy. These structural changes neither affected patient outcome nor impaired the ability to educate the surgical resident, but ensured a higher incidence of acute cases for the trainee to be involved in. The evidence reviewed in this article confirms international guidelines within the UK and should reassure all those involved that early cholecystectomy is indeed possible even with limited resources. NHS hospitals may need to set aside gallstone disease as a priority over other emergencies so that emergency theatre time may be ring-fenced for acute cholecystitis cases or establish rapid access multidisciplinary pathways to expedite appropriate patients to surgery.

While our synthesis supports ELC as the default for fit patients, older or highly comorbid patients may experience greater perioperative morbidity with emergency surgery, as reflected in some series included in the review, reinforcing the importance of individualised timing decisions.

Cost Effectiveness

Besides outcomes, there are economic arguments in favour of performing early cholecystectomy. We calculated that each extra bed-day costs an average of around £465 (based on the NHS England reference cost 2023/24) [26]. As such, any reduction in LOS, even one to two days per patient, can result in a huge amount of savings over thousands of cases. In addition, unplanned readmissions for biliary-related complications (e.g., recurrent cholecystitis or gallstone pancreatitis) are costly and have been estimated to cost between £1,200 and £1,800 per admission (depending on severity) [27]. This downstream cost can be saved to the NHS by preventing these occurrences with definitive early surgery. Extrapolating our results nationwide to an estimated 15,000 eligible cases of acute cholecystitis per annum, reduction of LOS and readmissions with the early surgery pathway may mean potential cost savings of about £14-28 million annually. Although back-of-the-envelope calculations are often misleading, there is an impression that investment in ELC (e.g., the funding of additional emergency theatre lists or staff) would pay for itself in the currency of lower bed occupancy and less emergency readmission. These economic benefits support the case for nationwide implementation of ELC as a new standard of care for acute calculous biliary disease in the UK.

Royal College of Surgeons (RCS) Cholecystectomy Quality Improvement Collaborative (Chole-QUIC) Initiative and Its Impact

The findings of this review resonate strongly with the goals and early outcomes of the Chole-QUIC initiative, which has demonstrated that sustainable system-wide change is not only possible but also highly impactful when early cholecystectomy is prioritised. As a clinician-led national quality improvement programme, Chole-QUIC targeted the systemic delays and operational inertia that have long impeded the timely surgical management of ACC in the NHS. Our synthesis of data from over 95,000 cases across the UK reinforces the core tenet of Chole-QUIC: that ELC, specifically within 72 hours of admission, yields better outcomes without increasing harm. Chole-QUIC’s own multi-centre evaluation found that hospitals participating in the programme improved the rate of index admission cholecystectomy from 24% to 42%, with no increase in complications, operative difficulty, or readmissions [28]. The broader evidence captured in this review mirrors and extends those results, supporting the view that early intervention should no longer be seen as aspirational or niche but as achievable best practice. Importantly, this review highlights the consistency of benefit across institution types, from high-volume tertiary centres to smaller district general hospitals. Such generalisability strengthens the argument that the challenges to early cholecystectomy are not inherent to patient complexity or surgical difficulty but lie instead in service design and resourcing, precisely the areas targeted by Chole-QUIC [28].

The barriers identified in this review, limited theatre capacity, rigid scheduling, and lack of prioritisation, were directly addressed through the collaborative's structured interventions, including the introduction of protected emergency theatre time, ‘hot gallbladder’ pathways, and consultant-led care models such as the “surgeon of the week”. These interventions echo broader surgical quality improvement principles: focusing on actionable change, iterative feedback, and frontline leadership.

The synergy between Chole-QUIC’s process redesign and the clinical outcomes summarised in this review provides a compelling blueprint for national scale-up. Indeed, the projected cost savings of £14-28 million per annum estimated in our analysis offer an economic imperative that aligns with ongoing efforts to improve NHS efficiency while preserving care quality. However, it is also notable that uptake of Chole-QUIC’s principles remains inconsistent. Despite robust data and successful pilots, variation in practice persists, with many trusts still defaulting to delayed or interval cholecystectomy, often citing logistical limitations. This inertia carries measurable clinical consequences: unnecessary morbidity from recurrent biliary events, increased healthcare utilisation, and lost opportunities for surgical training. In light of this evidence, we argue that the case for early laparoscopic cholecystectomy is now unequivocal, not just as a clinically safe intervention, but as an economically sound, ethically justified, and operationally viable standard. The Chole-QUIC initiative has laid the groundwork. The task now is to consolidate these gains and embed early cholecystectomy into national policy, commissioning frameworks, and surgical education as routine best practice.

## Conclusions

This UK-focused systematic review shows that ELC, when performed within the first few days of admission, is generally linked to shorter hospital stays, fewer readmissions, and similar or lower complication and conversion rates compared with delayed surgery or conservative management. UK data, from both single-centre audits and large cohort studies, indicate that operating during acute inflammation is safe and effective, challenging the traditional rationale for delay and highlighting the risks of recurrent biliary events, unplanned admissions, and technically more complex interval procedures.

In practice, ELC should be the default approach for most patients, but timing still needs to be individualised. Observational evidence suggests that older or high-risk patients may experience greater perioperative morbidity with emergency surgery, whereas a temporising strategy followed by interval cholecystectomy may be safer. Importantly, the NHS faces persistent barriers, such as limited emergency theatre time and variation in service delivery, that hinder timely surgery. Initiatives like protected emergency lists and dedicated “hot gallbladder” pathways show that these challenges can be overcome, supporting wider adoption of early surgery as a realistic and beneficial standard of care.

## References

[1] (2025). Hospital admitted patient care activity, 2022-23. https://digital.nhs.uk/data-and-information/publications/statistical/hospital-admitted-patient-care-activity.

[2] (2025). UK Surgical Workforce Census Report. RCS England.

[3] Gurusamy K, Samraj K, Gluud C, Wilson E, Davidson BR (2010). Meta-analysis of randomized controlled trials on the safety and effectiveness of early versus delayed laparoscopic cholecystectomy for acute cholecystitis. Br J Surg.

[4] (2025). Gallstone disease: diagnosis and management. https://www.nice.org.uk/guidance/cg188.

[5] Ansaloni L, Pisano M, Coccolini F (2016). 2016 WSES guidelines on acute calculous cholecystitis. World J Emerg Surg.

[6] Yokoe M, Hata J, Takada T (2018). Tokyo Guidelines 2018: diagnostic criteria and severity grading of acute cholecystitis (with videos). J Hepatobiliary Pancreat Sci.

[7] Cheruvu CV, Eyre-Brook IA (2002). Consequences of prolonged wait before gallbladder surgery. Ann R Coll Surg Engl.

[8] Page MJ, McKenzie JE, Bossuyt PM (2021). The PRISMA 2020 statement: an updated guideline for reporting systematic reviews. BMJ.

[9] Agrawal S, Battula N, Barraclough L, Durkin D, Cheruvu CV (2009). Early laparoscopic cholecystectomy service provision is feasible and safe in the current UK National Health Service. Ann R Coll Surg Engl.

[10] Cao AM, Eslick GD, Cox MR (2016). Early laparoscopic cholecystectomy is superior to delayed acute cholecystitis: a meta-analysis of case-control studies. Surg Endosc.

[11] Ouzzani M, Hammady H, Fedorowicz Z, Elmagarmid A (2016). Rayyan-a web and mobile app for systematic reviews. Syst Rev.

[12] Wells GA, Shea B, O'Connell D, Peterson J, Welch V, Losos M, Tugwell P (2021). The Newcastle-Ottawa Scale (NOS) for assessing the quality of nonrandomised studies in meta-analyses. Ottawa Hospital Research Institute.

[13] Saeb-Parsy K, Mills A, Rang C, Reed JB, Harris AM (2010). Emergency laparoscopic cholecystectomy in an unselected cohort: a safe and viable option in a specialist centre. Int J Surg.

[14] Young AL, Cockbain AJ, White AW, Hood A, Menon KV, Toogood GJ (2010). Index admission laparoscopic cholecystectomy for patients with acute biliary symptoms: results from a specialist centre. HPB (Oxford).

[15] Lucocq J, Patil P, Scollay J (2022). Acute cholecystitis: delayed cholecystectomy has lesser perioperative morbidity compared to emergency cholecystectomy. Surgery.

[16] Bekheit M, Rajan S, Wohlgemut JM, Watson AJ, Ramsay G (2023). Comprehensive assessment of the management of acute cholecystitis in Scotland: population-wide cohort study. BJS Open.

[17] Wiggins T, Markar SR, MacKenzie H (2019). Optimum timing of emergency cholecystectomy for acute cholecystitis in England: population-based cohort study. Surg Endosc.

[18] Hadad SM, Vaidya JS, Baker L, Koh HC, Heron TP, Hussain K, Thompson AM (2007). Delay from symptom onset increases the conversion rate in laparoscopic cholecystectomy for acute cholecystitis. World J Surg.

[19] Singhal T, Balakrishnan S, Grandy-Smith S, Hunt J, Asante M, El-Hasani S (2006). Gallstones: best served hot. JSLS.

[20] Down SK, Nicolic M, Abdulkarim H, Skelton N, Harris AH, Koak Y (2010). Low ninety-day re-admission rates after emergency and elective laparoscopic cholecystectomy in a district general hospital. Ann R Coll Surg Engl.

[21] Farooq T, Buchanan G, Manda V, Kennedy R, Ockrim J (2009). Is early laparoscopic cholecystectomy safe after the "safe period"?. J Laparoendosc Adv Surg Tech A.

[22] Vithayathil M, Yong C, Dawas K (2025). Clinical outcomes of early and delayed cholecystectomy for acute gallstone-related disease. Turk J Surg.

[23] Stephens MR, Beaton C, Steger AC (2010). Early cholecystectomy after acute admission with cholecystitis: how much work?. World J Surg.

[24] Petra G, Kritsotakis EI, Gouvas N (2025). Multicentre prospective study on the diagnostic and prognostic validity of malnutrition assessment tools in surgery. Br J Surg.

[25] Palomba G, Dinuzzi VP, Amendola A, Palomba R, DE Palma GD, Aprea G (2021). Laparoscopic cholecystectomy for acute cholecystitis: onset of symptoms and severity grade as a tool for choosing the optimal timing. Minerva Surg.

[26] (2025). 2023/24 National Cost Collection Data Publication. https://www.england.nhs.uk/publication/2023-24-national-cost-collection-data..

[27] Carter PR (2025). Operational productivity and performance in English NHS acute hospitals: unwarranted variations. https://assets.publishing.service.gov.uk/government/uploads/system/uploads/attachment_data/file/499229/Operational_productivity_A.pdf.

[28] (2025). Cholecystectomy Quality Improvement Collaborative (Chole-QuIC). https://www.rcseng.ac.uk/standards-and-research/standards-and-guidance/service-standards/emergency-surgery/cholecystectomy-quality-improvement-collaborative/.

